# Supplementary material for: Behavioral Correlates of COVID-19 Worry: Stigma, Knowledge, and News Source
Source: Int J Environ Res Public Health. 2021 Oct 30;18(21):11436. doi: 10.3390/ijerph182111436 (PMC8583421; doi:10.3390/ijerph182111436)
Supplement: Supplementary file 1 [file ijerph-18-11436-s001.zip › ijerph-1435765-supplementary.pdf]

Table S1. Phases of Data collection: Dates, Eligibility Criteria, Response Numbers, and Rationale.

	Phase	Start/End date	Inclusion/Exclusion criteria	Total Responses	Rationale
New York State Sample	1a. NYS Soft-Launch	07/02/2020 to 07/02/2020	(1) 18+ years of age, (2) New York State (NYS) resident, (3) previously completed at least 500 MTurk tasks; and (4) 90% approval ratings for previously completed MTurk tasks	*N*= 5	To test for any flaws in the survey logic or payment mechanism
	1b. NYS Launch	07/02/2020 to 08/01/2020	(1) 18+ years of age, (2) NYS resident; (3) previously completed at least 500 MTurk tasks; and (4) 90% approval ratings for previously completed MTurk tasks, and (5) did not complete phase 1 survey.	*N* = 597	After a period of diminished participation, it was determined that the slow response rate was attributed to limiting the participant pool to NYS residents.<br><br>As such, we re-launched the survey without the initial regional-filtering (phase 2).
National Sample	2. National Launch	08/05/2020 to 08/05/2020	(1) 18+ years of age; (2) previously completed at least 500 MTurk tasks; and (3) 90% approval ratings for previously completed MTurk tasks; and (4) did not complete phase 1 or 2a survey.	*N* = 605	
